# Retrospective Analysis of Serotype Switching of *Vibrio cholerae* O1 in a Cholera Endemic Region Shows It Is a Non-random Process

**DOI:** 10.1371/journal.pntd.0005044

**Published:** 2016-10-05

**Authors:** Stefan L. Karlsson, Nicholas Thomson, Ankur Mutreja, Thomas Connor, Dipika Sur, Mohammad Ali, John Clemens, Gordon Dougan, Jan Holmgren, Michael Lebens

**Affiliations:** 1 Department of Microbiology and Immunology, Institute of Biomedicine, University of Gothenburg, Gothenburg, Sweden; 2 Wellcome Trust Sanger Institute, Cambridge, United Kingdom; 3 Department of Pathogen Molecular Biology, the London School of Hygiene and Tropical Medicine, London, United Kingdom; 4 Cardiff University, Cardiff, United Kingdom; 5 Indian Council of Medical Research, New Delhi, India; 6 Johns Hopkins Bloomberg School of Public Health, Maryland, Baltimore, United States of America; 7 International Centre for Diarrhoeal Disease Research, Dhaka, Dhaka Bangladesh; Massachusetts General Hospital, UNITED STATES

## Abstract

Genomic data generated from clinical *Vibrio cholerae* O1 isolates collected over a five year period in an area of Kolkata, India with seasonal cholera outbreaks allowed a detailed genetic analysis of serotype switching that occurred from Ogawa to Inaba and back to Ogawa. The change from Ogawa to Inaba resulted from mutational disruption of the methyltransferase encoded by the *wbeT* gene. Re-emergence of the Ogawa serotype was found to result either from expansion of an already existing Ogawa clade or reversion of the mutation in an Inaba clade. Our data suggests that such transitions are not random events but rather driven by as yet unidentified selection mechanisms based on differences in the structure of the O1 antigen or in the serotype-determining *wbeT* gene.

## Author Summary

Cholera is a major health problem in many parts of the world causing seasonal outbreaks in endemic areas. Essentially only the O1 serogroup of *Vibrio cholerae* causes epidemic cholera. This serogroup has two immunologically distinguishable serotype variants called Ogawa and Inaba. The Inaba serotype is a consequence of a mutation in a single gene, *wbeT*, that in its intact form encodes for an enzyme that methylates the terminal perosamine sugar of the lipopolysaccharide side chain thus resulting in the Ogawa serotype. By careful examination over a five-year period of the genetic lineages of bacteria causing cholera in an endemic area we show data indicating that serotype switching is not a random process but is driven by selection pressures that have yet to be identified.

## Introduction

Cholera is the most severe of all infectious diarrheal diseases, which if not adequately treated can have a high mortality up to 50%. It is caused by *Vibrio cholerae* bacteria of serogroup O1, whose propensity for epidemic and even pandemic spread can have devastating effects in terms of morbidity and mortality. Recent examples of severe epidemics leading to many thousands of deaths are those that occurred in Zimbabwe and Haiti [1, 2]. Over the past nearly two centuries seven pandemics have been documented [3]. The first six pandemics are believed to have been caused by *V*. *cholerae* of the Classical biotype whereas the current ongoing pandemic that started in the early 1960’s is caused by organisms of the El Tor biotype [4]. Despite significant physiological and biochemical differences the two *V*. *cholerae* biotypes express almost identical virulence factors (including cholera toxin) and the same surface lipopolysaccharide (LPS). Both have the same O1 serogroup which, along with the relatively rarely seen serotype O139 (currently <0.1% globally and only occurring in South Asia), are the only *V*. *cholerae* known to cause epidemic or pandemic cholera [4].

In both biotypes the O1 serogroup can be further subdivided into two variants, serotypes Ogawa or Inaba. This phenotypic difference is due to the presence or absence of a methyl group on the terminal perosamine sugar of the surface LPS [5]. Isolates serotype as Ogawa if the O1 serogroup LPS is methylated and as Inaba if the LPS is not methylated [6]. Methylation is catalyzed by a methyltransferase encoded by the *wbeT* (formerly called *rbfT* [7]) gene and inactivating mutations in this gene result in the Inaba serotype. Both serotypes cause cholera outbreaks with no obvious differences in pathology [8]. Switching between Ogawa and Inaba serotypes has been noted previously in endemic regions [9–12] but the epidemiological significance of the two serotypic variants has hitherto remained largely uninvestigated and poorly understood, although it has been suggested that the serotype transition might be driven by acquisition of serotype-specific immunity within the host population [9].

In 2006 a large field trial in Kolkata, India was conducted to test the efficacy of an oral killed whole cell vaccine [13–15]. Cases of cholera were identified and closely monitored for 3½ years prior to and 1½ year following immunization. This included cataloguing every case of severe cholera (culture confirmed cases from patients seeking treatment and showing acute dehydration), defining the GPS coordinates of the dwelling of each patient, culturing and serotyping the responsible O1 *V*. *cholerae* and obtaining the complete genome sequence of almost 90% of all isolates collected. During this period there was a shift in the serotype of isolates causing disease from Ogawa to Inaba followed by a second shift back to Ogawa. The current paper describes the relationships between the organisms isolated from patients living in the Kolkata study area between 2003 and 2008. We used the genomic data from the isolated bacteria to determine their detailed relationships and genetic makeup. This allowed us to follow the emergence and fate of individual lineages giving new insights into the forces that may be driving the observed serotype transitions.

## Methods

### Selection of the catchment area, sampling, and serotype determination

The data presented in this paper are from positive diagnosis tests from cholera patients in a surveillance study 3 years prior to and two years after an oral killed whole cell cholera vaccine trial in the same area. The design of the vaccine trial including the choice of field site has been described previously and the trial is registered with ClinicalTrials.gov, number NCT00289224 [15]. The sampling, culturing and serotyping of the isolated organisms were done according to standard procedures [16].

### Whole genome sequencing and analysis of the wbeT gene

Genomic sequencing was done at the Sanger Institute using DNA isolated at NICED. Genomic libraries were prepared for each sample, followed by multiplex sequencing on an Illumina HiSeq. The 101–base paired–end reads obtained were mapped against the reference *V*. *cholerae* N16961 El Tor (AE003852 and AE003853), SNPs in the core genome were identified and these SNPs were used to generate a phylogeny based on the core genome as described previously [17].

To investigate the population structure of *V*. *cholerae*, we interpreted these chromosomal SNPs using Maximum likelihood (ML) phylogenetic analysis.

Once the genomic sequencing was done it became apparent that there were a small number of discrepancies between the serological data and the genomic data when ascribing Inaba or Ogawa serotypes (19 out of 411). For consistency we assigned serotype for all isolates included in this study based on the presence of an intact *wbeT* gene, by manual curation using the sequence of O1 *V*. *cholerae* El Tor strain VX44945 (JF284685) as reference. Strains with an intact *wbeT* and surrounding region were deemed to be Ogawa. Strains carrying mutations caused by insertion were deemed to be Inaba. Furthermore, strains with mutations in the *wbeT* gene that have been associated with the Inaba phenotype in the literature and shown to have the Inaba phenotype in our laboratory were also deemed to be Inaba. The term *geno–serotype* is used to describe the assignment of serotype based on the sequence of the *wbeT* gene. Strains with discrepancies in serotype assignment by the different methods were subsequently retested and it was found that in most cases the geno-serotype could be confirmed serologically.

### Ethical statement

The current paper uses data obtained from isolates of *Vibrio cholerae* cultured from patients seeking medical treatment due to diarrhea as a part of routine diagnostic procedures. The patients were resident within the catchment area for a clinical trial of an oral killed whole cell cholera vaccine [15] but not necessarily participants within the trial. Specific isolates can only be identified by collection date and GPS coordinates of patients’ residence and cannot be linked to individual patients. DNA was transferred from NICED to the Sanger Institute without any personal data from the patients from which the isolates were obtained.

## Results

### Regular seasonal cholera outbreaks in Kolkata municipal wards 29, 30 and 33 in the period 2003–2008

**Fig 1:**
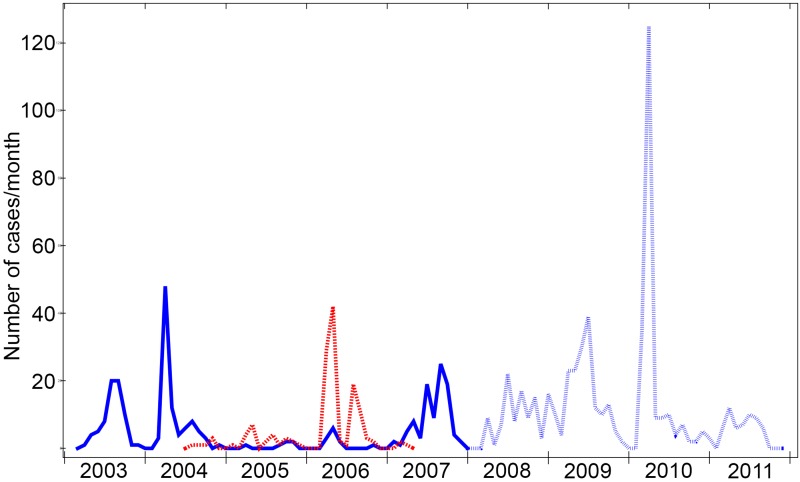
Cholera cases recorded in municipal wards 29, 30 and 33 in Kolkata. All cases of cholera showing serotype of isolated strains, Ogawa (blue solid line) and Inaba (red dotted lined), over the period from March 2003 until the end of 2007, shown per month. The blue dashed line shows the cases recorded over an extended period within the same area of Kolkata until the end of 2011 [18]. There are generally seasonal outbreaks with one in the spring and one in the autumn.

**Fig 2:**
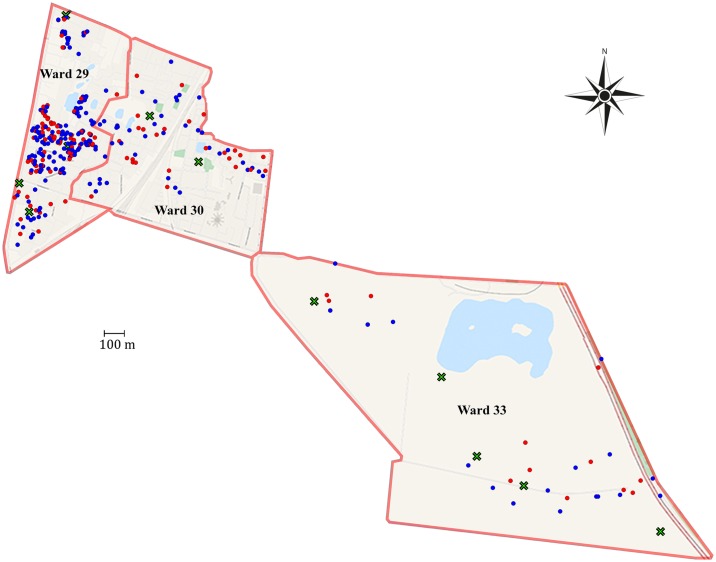
The distribution of cholera cases. The three wards of Kolkata (29, 30 and 33) in which the study was conducted showing all the documented cases of cholera according to the GPS coordinates of the patients. Green X represents project health clinics, blue and red dots represent Ogawa and Inaba serotyped cases respectively.

Cholera outbreaks occurred seasonally in each of the years defining the study period, usually in two waves between March and September (Fig 1). A total of 462 *V*. *cholerae* isolates, all of them being typed as O1 El Tor, were collected from patients presenting with acute diarrhoea for 3½ years prior to and 1½ year following the first vaccination in the clinical trial in June 2006 (spanning the period from January 2003 to December 2007) in Kolkata municipal wards 29, 30, and 33 (Fig 2). Throughout the study the cholera cases from which *V*. *cholerae* were isolated are referred to as “severe” since the disease symptoms caused the patients to seek treatment at a health care center.

The cholera cases were widely distributed over the wards, with the greatest concentration being in ward 29 (Fig 2). Overall in 2005 relatively few cholera cases were reported, amounting to only 13% of the cases compared to the preceding and following years. When the period of observation is extended until 2011 using published data from You *et al* [18] for the same area (Fig 1), it can be seen that also in 2011 the occurrence of cases is unusually low and that this again follows a large outbreak with a lot of cases in a short period in 2010.

### Both serotype Inaba and Ogawa cholera outbreaks occurred during the study period

Looking at the temporal distribution of the isolates causing disease it is clear that both Inaba and Ogawa isolates were responsible for causing intermittent outbreaks in this area across the whole study period. However, it is also apparent that the peaks of infection were dominated by either one or the other serotype (Fig 1). When Ogawa infections were dominant in general no Inaba strains were isolated except in the transition period in 2004 when Inaba strains began to emerge as clinical isolates and in 2007 when the number of Inaba isolates was in sharp decline. Thereafter there were no recorded Inaba cases in cholera patients for at least the following five years [18]. In contrast, during the two-year period 2005–06 when Inaba predominated Ogawa strains were also isolated (Fig 1). There was no perceptible geographical demarcation between the Ogawa and Inaba serotype strains (Fig 2)

### Phylogenetic relationships in the temporal distribution of Ogawa/Inaba isolates

Of the 462 *V*. *cholerae* isolates collected, 411 were successfully genome sequenced. Sequencing probably failed in the remainder of the samples due to poor DNA quality resulting from degradation in transit from India to the UK.

**Fig 3:**
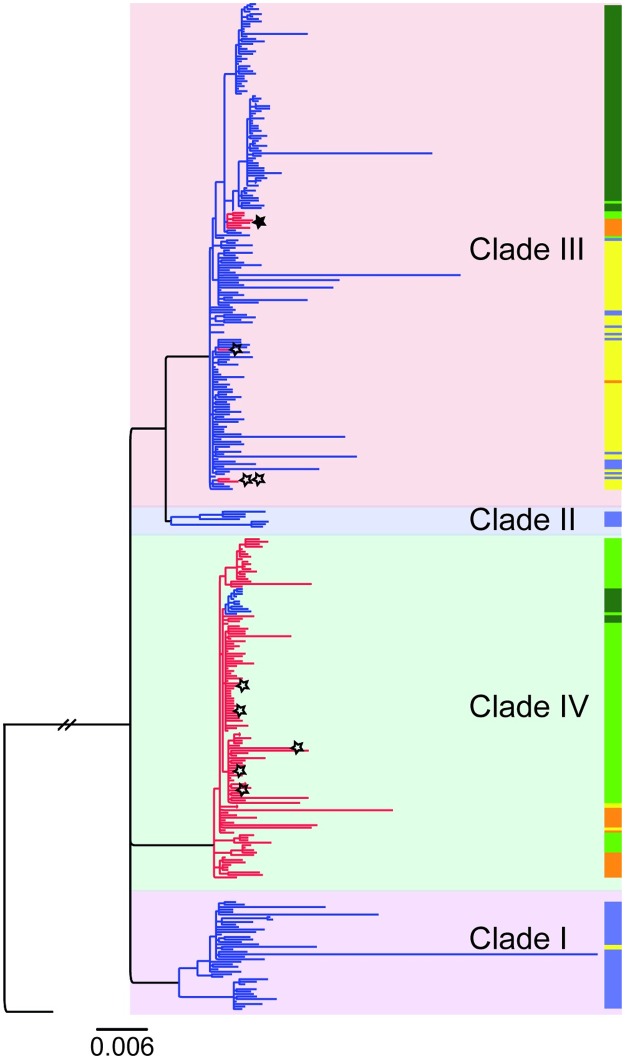
Phylogenetic relationships showing four distinct clades. A phylogenetic tree summarizing the data accumulated in the described study indicating the clade, serotype and year of isolation. It can be seen that the strains cluster by clade regardless of which of the criteria is used. There is however a striking correlation between the year of isolation and serotype. Key: Blue and red lines in the tree indicate serotype; Ogawa and Inaba respectively. The colored bar indicates the year of isolation; light blue = 2003, yellow = 2004, orange = 2005, light green = 2006, and dark green = 2007. The stars on the tree indicate strains in which inserts in the *wbeT* gene or mutations other than S158P - have been identified. The filled star indicates Inaba strains in Clade III resulting from a clonal expansion of an Inaba strain with an insertion in the *wbeT* gene. The additional Inaba strains in Clade III result from an M1I substitution (indicated by two stars).

**Fig 4:**
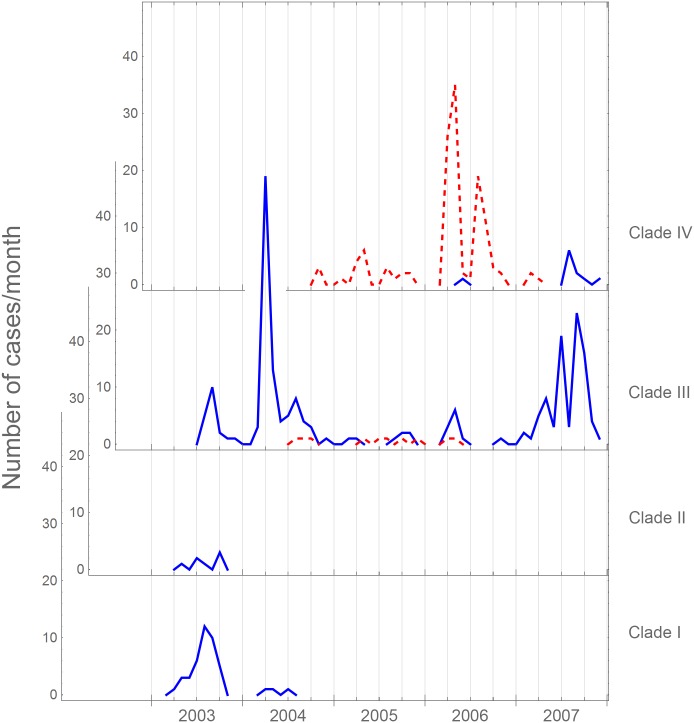
Timeline of all strains according to clade. Diagram showing the total number of cholera cases that occurred in the surveyed area during the period from March 2003 to December 2007 divided to show the time line for each of the four identified clades. The blue (solid) lines represent Ogawa strains and the red (dotted) lines represent Inaba strains.

The genomic analysis showed that all sequenced isolates fall within the previously described “wave three” of the global pandemic 7 lineage of *V*. *cholerae* biotype El Tor [17]. The isolates could be divided into four clades on the basis of the clustering pattern in the phylogenetic tree as indicated in Fig 3. In general, definable outbreaks could be explained by the expansion of one predominant clade, even though there were usually bacteria from at least two clades causing disease during any outbreak period (Fig 4). Clade III isolates were predominantly Ogawa with a few sporadic Inaba strains isolated from 2004 to 2006. Isolates from Clade IV were predominantly Inaba first appearing in 2004 although a number of Ogawa strains from this clade were also isolated in 2006 and 2007.

### Mutations in wbeT causing the Inaba phenotype are not uniform

**Fig 5:**
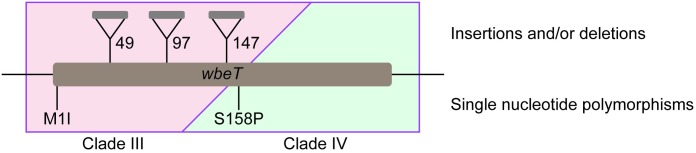
Schematic diagram of the *wbeT* gene showing mutations giving rise to the Inaba serotype in the current study. The insertions and the M1I mutations were confined to Clade III and the S158P mutation was confined to clade IV. In all there were 219 isolates in Clade III. The insertion at amino acid position 49 occurred in seven isolates and the insertions at 97 and 147 were present in a single isolate. Two strains were isolated with the M1I mutation. The remaining 209 isolates were Ogawa. In Clade IV there were 139 isolates. Of these 128 were Inaba due to the S158P mutation and 11 were Ogawa due to a reversion event. 5 out of the 128 Inaba isolates in Clade IV carried multiple insertions in addition to the S158P mutation (not shown).

Sequence analysis of the *wbeT* gene, whose inactivation is responsible for the Inaba serotype classification, showed that across the Inaba isolates there were multiple different mutations present within this gene that could explain the inactivation of WbeT methylation activity (Fig 5). Within Inaba isolates of Clade III (associated with a minority of the isolated cases of Inaba cholera) two types of mutation were seen: either a nonsynonymous single base substitution (M1I) that disrupts the initiation of translation, or insertion elements (similar to SXT IS4 family transposase gene [gb|KC709654.1|] or *Vibrio cholerae* 569b mega-integron [gb|AF179596.1|]) that disrupted the gene by introducing a stop codon. In Clade IV, (associated with the large Inaba outbreaks of 2006) all of the 128 Inaba isolates had an identical SNP(ACT→CCT) resulting in an amino acid substitution, S158P in the translated protein leading to inactivation with respect to methylation of LPS; five of these strains also carried insertion elements similar to *Vibrio cholerae* 569b mega-integron (gb|AF179596.1|) and/or IS1358 (gb|U93589.1|) in the *wbeT* gene.

### Clade IV isolates persisting after 2006 had the Ogawa serotype

The Inaba outbreaks in 2006 were caused almost exclusively by Clade IV. Strikingly however, although the majority of this clade carry an inactivating mutation in *wbeT* a cluster of Clade IV Ogawa isolates emerged that had apparently reverted to the wild type *wbeT* gene by the loss of the non-synonymous SNP (ACT→CCT resulting in the S158P mutation). The first Ogawa revertant appeared in June of 2006. More than a year later, in the autumn of 2007, the strain had expanded and several more Ogawa cases from the same lineage were isolated. At this time no Clade IV Inaba strains were isolated from cholera patients (Fig 4). Thus in 2007 the Ogawa serotype re-emerged as the sole cause of cholera due to the expansion of two distinct lineages (Clades III and IV) one of which arose as the result of a reversion mutation of an Inaba strain from Clade IV.

## Discussion

The presented data represents a detailed whole genome-based dissection of *V*. *cholerae* O1 isolates causing repeat outbreaks of cholera in Kolkata, India over a five-year period. The results show clearly the emergence and subsequent disappearance of Inaba serotype isolates in an endemic area where the Ogawa serotype predominates as the cause of endemic cholera.

The generally accepted view of cholera outbreaks is that in most circumstances there is an index case that sheds large numbers of virulent bacteria into the environment contaminating food and water-sources and also spreading infection by family contacts [19]. In non-endemic areas this is likely to be the case since cholera is introduced into an area where it was previously absent. However, the presented data show that in a highly endemic area such as the one studied in Kolkata there are several closely related clades present of 7^th^ pandemic O1 *V*. *cholerae*, all of which cause cholera even though expansions of individual clades account for most of the cases in any particular outbreak. The state of flux of the different clades in the area is evidenced by the disappearance of Clades I and II which ceased to be isolated after 2004, and the persistence of Clade III as a cause of cholera throughout the period.

The data shows that there was a clear point where there was a change in the serotype of the organisms causing clinical cholera from exclusively Ogawa towards predominantly Inaba culminating in major Inaba outbreaks in 2006. These outbreaks can be attributed to a clonal expansion of Clade IV. However, the emergence and continued presence of two lineages carrying different mutations leading to the same phenotype (non-methylated LPS serotyping as Inaba) emerging at almost the same time suggests that the phenotype itself was of importance for the persistence of these strains during this period and that the transition from Ogawa to Inaba cannot be explained by an Inaba index case. Indeed, it is notable that Clade III Inaba strains were the first to be isolated but did not expand to a significant extent compared to Clade IV Inaba strains that were first isolated three months after the first Inaba isolates appeared in 2004 and yet did not cause significant outbreaks until 2006. Whether this was due to conditions not being conducive to a cholera outbreak until 2006 or whether it reflects a difference in the ability of the different Inaba strains to expand and persist is open to question.

The disappearance of Inaba isolates from patients in contrast was abrupt and also characterized by the emergence of two lineages. Clade III Ogawa isolates again predominated, but there was also an expansion of a Clade IV lineage in which there was a reversion of the *wbeT* gene back to the active form. There were no Inaba strains from Clade III isolated after the first half of 2007 and, although there is no genomic data after 2007, no Inaba strains were isolated in the area during the next five years of follow-up.

It has previously been shown that the core genome of *Vibrio cholerae* accumulates approximately 3.3 SNPs per genome per year [17]. The *wbeT* gene in Ogawa strains is highly conserved with no synonymous SNPs observed either in published *wbeT* from El Tor O1 *V*. *cholerae* sequences from GenBank, in our own large geographically and temporally diverse strain collection (unpublished results) or in the current study (273 isolates). However, in the current study despite the low overall diversity between the isolates, in isolates of the Inaba serotype two different SNPs and at least 7 independent insertion events in the *wbeT* gene were observed in less than three years. It is unlikely that the near-simultaneous emergence of multiple lineages with different mutations in the *wbeT* gene leading to the same phenotypic change was a random event. It seems more likely that specific non-random triggers precipitated both the emergence and subsequent disappearance of the Inaba isolates. At present the nature of such trigger(s) is unclear. It has been suggested that expansion of vibriophage populations is a factor that limits cholera outbreaks [20]; hence the presence of a serotype-specific phage could possibly explain the decline in Ogawa isolates and the emergence of insensitive Inaba strains and vice versa. The sudden decline in the number of Ogawa cases and the first relatively slow emergence of Inaba could reflect initial selection against Ogawa isolates combined with a low number of Inaba strains in the environment.

These studies show that the *wbeT* gene is highly conserved yet under certain circumstances inactivating mutations are readily isolated by what appears to be a driven process. The overall strong conservation of the *wbeT* gene suggests that it has an important role whereas the ease with which mutants can be isolated at times suggests that under certain conditions the change to Inaba may contribute to the ability of *V*. *cholerae* O1 to persist in an endemic environment.

Although the presented study provides a detailed picture of strains causing severe cholera over time in an area where the disease is endemic, it is also limited. Only samples from patients with clinical disease were analyzed representing only a fraction of all cholera strains in the study population since the majority of cholera infections result in milder symptoms or are asymptomatic [21]. We also have no data concerning environmental bacterial populations. Furthermore, the data was collected in association with a clinical trial of a cholera vaccine and whereas the first serotype transition occurred before the first vaccinations in 2006 and can have had no influence on the initial Ogawa to Inaba serotype shift, some effect of the vaccination on the subsequent transition back to Ogawa cannot be ruled out.

Nonetheless, the detailed data are able to differentiate lineages in a way that provides a valuable novel insight into their persistence and spread in an endemic area and suggest that the dynamics are more complex than previously thought. Furthermore, although no comparable studies have been done, the occurrence of the same sorts of mutation in the *wbeT* gene from isolates from different outbreaks in different parts of the world suggest that our observations do not represent a unique event [22–26].

Clearly further work needs to be done to address the factors contributing to the persistence and spread of cholera in endemic areas. However, our data suggests that shifts in serotype of O1 *V*. *cholerae* causing cholera are being driven by as yet undetermined non-random mechanisms and that mutations in the *wbeT* gene associated with serotype switching could be of importance for persistence of cholera in endemic areas. It remains to be tested whether it is the serotype shift alone that is important in this regard or whether the *wbeT* gene might have additional function; it is notable that Inaba strains isolated during outbreaks both in the present study and from elsewhere characteristically display single-point mutations in *wbeT* allowing for a full-length protein product in contrast to the Inaba isolates from the sporadic (non-epidemic) cholera cases in the present study that without exceptions had *wbeT* mutations incompatible with full-length protein translation.
